# Nanotechnology in cancer diagnosis: progress, challenges and opportunities

**DOI:** 10.1186/s13045-019-0833-3

**Published:** 2019-12-17

**Authors:** Ye Zhang, Maoyu Li, Xiaomei Gao, Yongheng Chen, Ting Liu

**Affiliations:** 1 Department of Oncology, NHC Key Laboratory of Cancer Proteomics, XiangYa Hospital, Central South University, Changsha, 410008 China; 2 Department of Gastroenterology, XiangYa Hospital, Central South University, Changsha, 410008 China; 3 Department of Pathology, XiangYa Hospital, Central South University, Changsha, 410008 China

**Keywords:** Nanoparticle, Cancer diagnosis, Cancer biomarker

## Abstract

In the fight against cancer, early detection is a key factor for successful treatment. However, the detection of cancer in the early stage has been hindered by the intrinsic limits of conventional cancer diagnostic methods. Nanotechnology provides high sensitivity, specificity, and multiplexed measurement capacity and has therefore been investigated for the detection of extracellular cancer biomarkers and cancer cells, as well as for in vivo imaging. This review summarizes the latest developments in nanotechnology applications for cancer diagnosis. In addition, the challenges in the translation of nanotechnology-based diagnostic methods into clinical applications are discussed.

## Background

Throughout the world, cancer mortality and incidence are increasing. As estimated by GLOBOCAN 2018, the number of new cancer cases will reach 18.1 million, and the number of cancer-related deaths will be 9.6 million [1, 2]. Predictions suggest that by 2030, 30 million people will die from cancer each year [2]. In the fight against cancer, a key for successful cancer treatment is early detection. Cancer-related mortality is greatly reduced by early detection [3]. For example, breast cancer exhibits a 5-year relative survival rate of nearly 90% at the local stage, while patients with distant metastasis exhibit a 5-year survival rate of only 27% [4].

At present, imaging techniques and morphological analysis of tissues (histopathology) or cells (cytology) aid in early diagnosis of cancer. The most widely used imaging techniques, such as X-ray, magnetic resonance imaging (MRI), computed tomography (CT), endoscopy, and ultrasound, can only detect cancer when there is a visible change to the tissue [5]. By that time, thousands of cancer cells may have proliferated and even metastasized. In addition, current imaging methods cannot distinguish benign lesions from malignant lesions [6]. Moreover, cytology and histopathology cannot be effectively and independently applied to detect cancer at an early stage [7]. Therefore, the development of technologies for detecting cancer at an early stage, before metastasis, presents a major challenge.

**Fig. 1:**
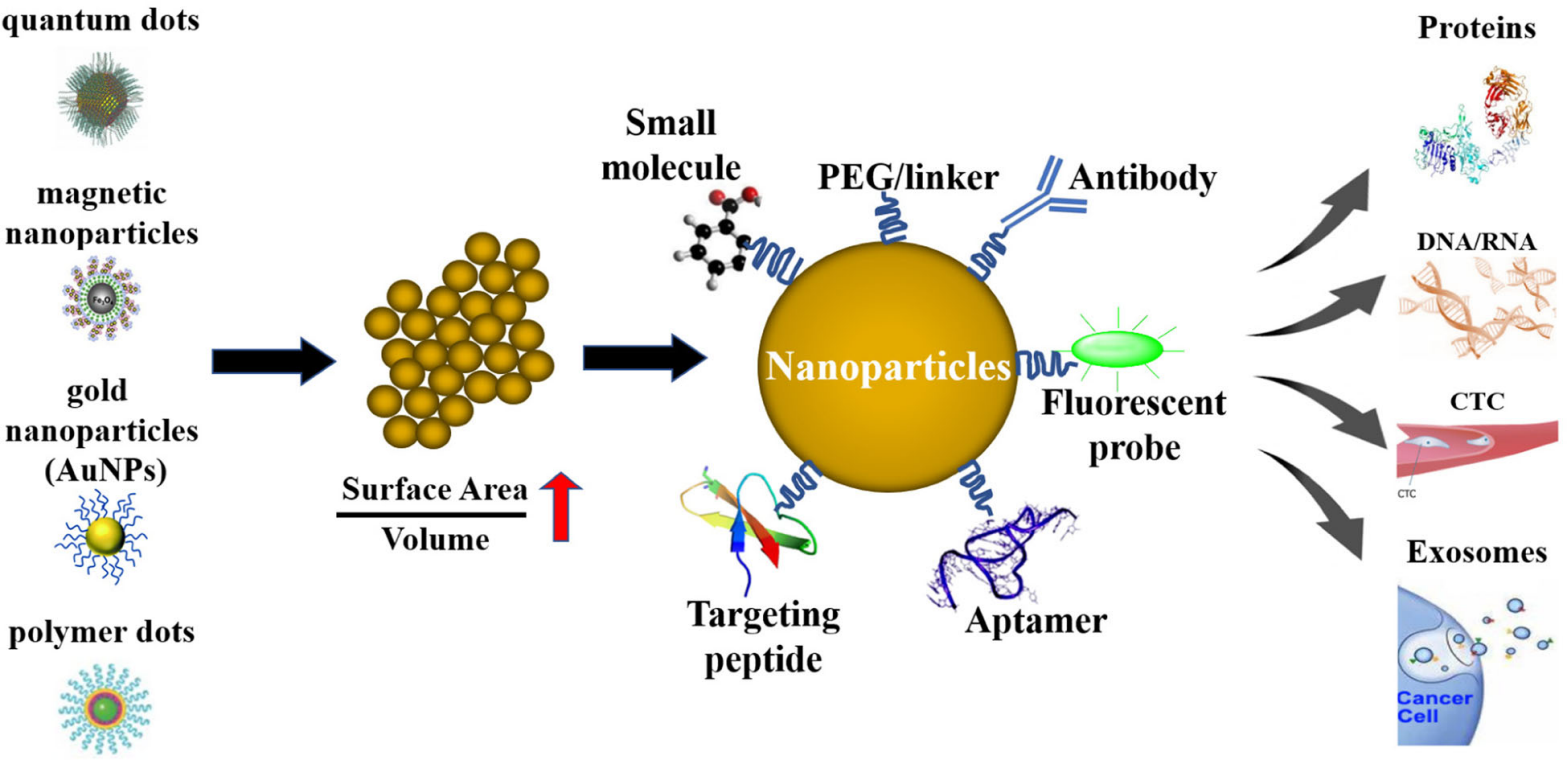
Nanotechnology improves cancer detection and diagnosis

Although nanotechnology has not yet been deployed clinically for cancer diagnosis, it is already on the market in a variety of medical tests and screens, such as the use of gold nanoparticles in home pregnancy tests [8]. For cancer diagnosis, nanoparticles are being applied to capture cancer biomarkers, such as cancer-associated proteins, circulating tumor DNA, circulating tumor cells, and exosomes [9]. An essential advantage of applying nanoparticles for cancer detection lies in their large surface area to volume ratio relative to bulk materials [10]. Due to this property, nanoparticle surfaces can be densely covered with antibodies, small molecules, peptides, aptamers, and other moieties. These moieties can bind and recognize specific cancer molecules (Fig. 1). By presenting various binding ligands to cancer cells, multivalent effects can be achieved, which can improve the specificity and sensitivity of an assay [11].

Nanotechnology-based diagnostic methods are being developed as promising tools for real-time, convenient, and cost-effective cancer diagnosis and detection [12]. This review summarizes recent progress in the development of nanotechnology and addresses the application of nanotechnology in cancer diagnosis. We also provide our perspective on challenges in the use of nanotechnology for cancer diagnosis.

### Nanotechnology for the detection of extracellular cancer biomarkers

A cancer biomarker acts as a measurable biological molecule that can be found in blood and other tissues or body fluids, such as saliva and urine, indicating that cancer exists in the body [13, 14]. Cancer biomarkers may be proteins (secreted proteins or cell surface proteins) [15], carbohydrates [16], or nucleic acids (circulating tumor DNA, miRNA, etc.) [17] that are secreted by the body or cancer cells when cancer is present [18, 19]. The measurement of certain cancer biomarker levels enables early detection of cancer or tumor recurrence and helps monitor the efficacy of the therapy. Nevertheless, the use of biomarkers has been limited by several barriers, including low biomarker concentrations in body fluids, heterogeneity in the abundance and timing of biomarkers within patients, and the difficulty in carrying out prospective studies [20]. Nanotechnology offers high selectivity and sensitivity and the ability to conduct simultaneous measurements of multiple targets. Biosensors can be improved with nanoparticles/nanomaterials to provide specific targeting [21]. In addition, the use of nanoparticles provides an increased surface-to-volume ratio, which makes biosensors more sensitive in fulfilling the demands of specific biomolecular diagnostics [22]. Quantum dots (QDs), gold nanoparticles (AuNPs), and polymer dots (PDs) are three common nanoparticle probes used in diagnosing cancer [23, 24].

#### Protein detection

A number of proteins have been granted FDA clearance for cancer detection, including CEA (colorectal cancer), AFP (liver cancer), PSA (prostate cancer), and CA-125 (ovarian cancer). Specific interactions with antibodies, antibody fragments, or aptamers can help in the detection of these properties. The interaction event will then be converted into a quantifiable signal that can be measured [25].

In recent studies, QD-based biosensors have been used for detecting cancer biomarkers. QDs are characterized by a high quantum yield and molar extinction coefficient; wide absorption with narrow, high-efficiency Stokes shifts; high resistance to photobleaching; and outstanding resistance to degradation, which constitute unique properties [26, 27]. A sandwich-type assay is a common strategy for detecting protein biomarkers and comprises many components, namely, a biomarker, a capture antibody, a second capture antibody, and a secondary antibody that binds to the capture antibody [7]. The secondary antibody can be visualized through various methods, such as staining and fluorescence [28].

In utilizing this strategy, two QD-conjugated antibodies against neuron-specific enolase (NSE) and carcinoembryonic antigen (CEA) were used to detect two biomarkers, and the limit of detection (LOD) of each reached 1.0 ng/ml [29]. A zinc oxide (ZnO) QD-based sandwich immunoassay was developed for ZnO nanowire substrates, which provided a large surface area that presents multiple binding sites used for detection. CEA, the most popular cancer biomarker, has been applied for monitoring of anticancer treatment, as well as for prediction of tumor recurrence following surgical resection in late-stage cancer patients, making it widely studied. NSE is an enzyme that catalyzes the conversion of 2-phosphoglycerate to phosphoenolpyruvate, which shows a relationship with carcinoids, small cell lung carcinoma, and islet cell tumors. After secretion, they could be detected at concentrations over 15 ng/mL, and the LOD of each reached 1.0 ng/mL. Another example of an immunosensor based on QDs is ZnO QDs coated with antibody against carbohydrate antigen 19-9 (CA 19-9, a biomarker for pancreatic ductal adenocarcinoma), which is an important application in the detection of CA 19-9. Electrostatic adsorption primarily aided in the immobilization process on the basis of the high isoelectric point of ZnO, and the immune reaction of the CA 19-9 antigens and antibodies produced the sandwich structure. CA 19-9 immunological recognition was converted into the detection of amplified signals presented by square wave stripping voltammetry (SWV), as well as the inherent photoluminescence (PL) exhibited by the labeled QDs. The dynamic range of the electrochemical assay was 0.1–180 U/ml, and the LOD reached 0.04 U/ml, while the dynamic range exhibited by the optical spectral detection was 1–180 U/ml, and the LOD reached 0.25 U/ml [30].

Peptides are frequently applied to actively target cancerous tissues in vivo [31]. The Arg-Gly-Asp (RGD) peptide motif is recognized by a receptor (integrin αvβ3) on the cell surface implicated in cancer metastasis and angiogenesis and has been applied to target tumor tissue in vivo for diagnosis [32]. In one study, an iRGD (CRGDKGPDC)-mediated and enzyme-induced precise targeting gold nanoparticle system (iRGD/AuNPs-A&C) was developed by simply co-administering the tumor-homing penetration peptide iRGD with a legumain-responsive aggregable gold nanoparticle [33]. iRGD/AuNPs-A&C showed high penetration and accumulation in 4 T1 mammary tumors [34].

Aptamers, which are single-stranded DNA (ssDNA) or RNA sequences that can be isolated via exponential enrichment (SELEX) that relies on ligand systematic evolution, can also be conjugated to nanoparticles [35, 36]. Aptamers have high affinity and strong binding specificity for their respective targets, such as ions, bacteria, peptides, viruses, phospholipids, and even whole cells. A10 RNA aptamer-conjugated polymeric nanoparticles incorporating Cy5 can bind to prostate-specific membrane antigen (PSMA). Cy5-PLA/aptamer NPs could only bind to LNCaP cells and canine prostate adenocarcinoma cells, which are positive for PSMA but not to PC3 cells, which are negative for PSMA. Cy5-PLA NPs have been applied in balb/c mice and exhibit outstanding signals with low-background fluorescence in different organs [37].

Rare-earth upconverting nanophosphors (UCNPs) promise to be a new generation of biological luminescence labels. UCNPs are able to absorb radiation from near-infrared (NIR) light and transform the radiation into visual light by relying on the upconversion process after multiple-photon absorption. Overexpression of secreted phospholipase A2 (sPLA-2), an enzyme that catalyzes phospholipid hydrolysis, has been reported to show an association with prostate cancer cell proliferation. Similar to phospholipids, phosphate surfactants are beneficial for recognizing and cleaving sPLA-2 enzyme. This unique characteristic could substantially help control UCNP release, because the enzyme hydrolyzes an ester group located between the fatty acid and ethylene glycol, which instantly liberates the nanoparticles from the prostate cancer surface [38].

#### ctDNA detection

Circulating tumor DNA (ctDNA) represents tumor-derived DNA fragments (approximately 100-200 base-pairs long) in the bloodstream [39]. ctDNA can be released from primary tumors or circulating tumor cells (CTCs) and can allow detection of cancer through cancer-specific genetic aberrations. Detection of genetic aberrations in ctDNA can help detect cancer even before any sign of cancer occurs [40, 41]. Highly specific hybridization with nucleic acid probes that have complementary sequences can be used to detect cancer-associated genetic aberrations [35]. A DNA silver nanocluster (NC) fluorescent probe was developed for detection of a single exon in the BRCA1 gene in breast cancer [42]. Under optimized conditions, this probe increased the LOD to 6.4 × 10^-11^ M. Large deletion mutations in BRCA1 were detected based on nanocluster fluorescence upon hybridization induced by recognition. The certain hybridization of the DNA-templated silver NC fluorescent probe to target DNAs was able to effectively enhance the AgNC fluorescence, which had various intensities, thereby distinguishing the BRCA1 deletion. When conditions were optimal, there was an increase in the fluorescence intensity presented by DNA-AgNCs at emission peaks near 440 nm (excitation at 350 nm) as the presence of the deletion type increased in a dynamic range of 1.0 × 10^-10^ to 2.4 × 10^-6^ M, and the LOD reached 6.4 × 10^-11^ M. In this sensing system, the deletion type led to higher fluorescence emission than the normal type, with the normal type typically showing low fluorescence.

#### microRNA detection

microRNA is associated with cancer diagnosis. Jou AF reported a two-step sensing platform for sensitive detection of miR-141, a promising biomarker for prostate cancer. The first step of the sensing platform used CdSe/ZnS QDs modified with FRET quencher-functionalized nucleic acids, which contained a telomerase primer sequence together with a recognition sequence for the miR-141 recognition sequence. The FRET quencher exhibited covalent binding to the nucleic acid-functionalized CdSe/ZnS QDs. When miR-141 hybridized with the probe, a duplex was formed, which would be cleaved by duplex-specific nuclease (DSN). The cleavage released the quencher unit and activated the fluorescence of the QDs. This cleavage also led to exposure of the telomerase primer sequence. The second step involved the primer unit elongation stimulated by telomerase/dNTPs, incorporation of hemin, and chemiluminescence generated with the help of luminol/H_2_O_2_. This platform helped detect miR-141 in a serum sample and discriminated healthy individuals from prostate cancer carriers [43].

#### DNA methylation detection

The genome methylation landscape (Methylscape) was recently reported as a common characteristic of most types and cancers and therefore could be a common cancer biomarker [44]. In this study, the authors observed differences between cancer genomes and normal genomes based on DNA-gold affinity and DNA solvation and developed simple, quick, selective and sensitive electrochemical or colorimetric one-step assays to detect cancer.

#### Extracellular vesicle detection

As circulating vesicles (30 nm–1 μm), extracellular vesicles (EVs) package molecular information, such as miRNA, DNA, protein, and mRNA, from mother cells and allow the detection of the molecular state of tumor cells that are difficult to access. In a recent study, the authors developed a new magnetic nanopore capture technique to isolate certain subsets of extracellular vesicles (EVs) from plasma [45]. The machine-learning and RNA-sequencing algorithms helped identify EV miRNA biomarkers. This approach was applied to a mouse model of pancreatic ductal adenocarcinoma (PDAC) and contributed to identification of a biomarker panel of eleven EV miRNAs.

Recently, Tan et al. reported a sensor platform that profiles proteins on the surface of exosomes within several minutes. The sensor consists of a gold nanoparticle (AuNP) and an aptamer panel under complexation, which was designed based on 13-nm AuNPs that were noncovalently conjugated with a panel of 5 aptamers that targeted cell surface proteins with high affinity and strong specificity, as demonstrated previously. The aptamers complexed with AuNPs prevented nanoparticle aggregation in a solution with high salt. Exosomes helped break the weak and nonspecific binding between the AuNP and aptamers, while strong and specific binding between aptamers and exosome surface proteins displaced aptamers from the AuNP surface, thereby facilitating AuNP aggregation. Due to aggregation, the color of the AuNPs changed from red to blue, indicating that the aptamers were bound to exosomal proteins. The intensity presented by the AuNP aggregation (A650/A520) was indicative of the relative abundance exhibited by target proteins on the surface of exosomes [46].

### Nanotechnology for detection of cancer cells

#### Detection of circulating tumor cells

Approximately 90% of deaths from solid tumors are attributed to metastasis [47]. In the course of metastatic dissemination, a cancer cell from the primary tumor first invades the surrounding tissue and then enters the microvasculature of the blood (intravasation) and lymph systems, followed by survival and translocation through the bloodstream to microvessels in distant tissues, subsequent exit from the bloodstream (extravasation) and survival in the microenvironment of distant tissues, which present a suitable foreign microenvironment for development of a macroscopic secondary tumor [48]. Early detection of metastatic cancer cells in the bloodstream, also known as circulating tumor cells (CTCs), can potentially affect cancer prognosis and diagnosis.

As a portion of a liquid biopsy, CTCs have been studied broadly due to their potential applications. CTC detection can help us understand the molecular organization of a tumor in a minimally invasive manner. Nevertheless, CTCs exhibit relatively low abundance and heterogeneity, which presents technical challenges for CTC isolation and characterization. In recent years, researchers have focused on the application of nanotechnologies for sensitive detection of CTCs; these technologies can help characterize cells and molecules, thereby enjoying broad clinical applications, such as disease detection at an early stage and evaluation of the treatment response and disease development.

As demonstrated in many studies, it is possible for cell pseudopodia to form on surfaces with nanostructure, thereby enhancing the local topographical interactions between cancer cells and nanostructured substrates, which is beneficial to CTC enrichment. For CTC detection, nanomaterials have an essential advantage in their large surface-to-volume ratio, which enables adsorption of high-efficiency targeting ligands with the ability to recognize specific molecules on cancer cells; therefore, CTC isolation shows high specificity and recovery, and the detection sensitivity is enhanced.

People have reported different types of nanomaterials, such as magnetic nanoparticles (MNPs), gold nanoparticles (AuNPs), quantum dots (QDs), nanowires, nanopillars, silicon nanopillars, carbon nanotubes, dendrimers, graphene oxide, and polymers, for CTC detection (Table 1) [61]. It has been demonstrated that these nanomaterials can improve the sensitivity and specificity of CTC capture devices and have the potential to facilitate cancer diagnosis and prognosis.

**Table 1:** Nanotechnology for detection of cancer cells

Nanoparticle	Type of affinity probe	Specificity ligand	Type of cancer	Reference
Magnetic nanoparticles	Antibody	EpCAM	Colon/liver /lung/breast	[49–51]
Quantum dots	Aptamer	PTK7	Leukemia	[52]
Polymer dots	Antibody	EpCAM	Breast	[53]
Gold nanoparticles	Aptamer Antibody	Her2 Cd2/cd3	Breast Leukemia	[54] [55]
Carbon nanotubes	Antibody	EpCAM	Liver	[56]
Upconversion NPs	Antibody	Her2	Breast	[57]
Nanorod arrays	DNA aptamer	EpCAM	Breast	[58]
Nanoparticle-coated silicon bead	Antibody	EpCAM/CD146	Breast Colorectal	[59]
Nanofibers	Antibody	EpCAM	Breast	[60]

In the field of nanobiotechnology, MNPs are mature nanomaterials that can bind to cells and have long been used for in vitro separation with the help of an external magnetic field [62]. Antibody-functionalized MNPs, namely, immunomagnetic nanoparticles, are frequently applied in the biomedical field. For CTC detection, immunomagnetic technologies usually specifically target EpCAM-expressing CTCs with anti-EpCAM functionalized MNPs.

To perform single-cell transcriptional profiling of CTCs purified from breast cancer patients, Powell et al .[63] used MagSweeper, which is an immunomagnetic enrichment device that can isolate tumor cells from unfractionated blood. MagSweeper serves as a magnetic cell sorting system that uses magnetic rods covered by a sheath to sweep across capture wells and attract target cells labeled with magnetic nanoparticles [64]. It can be used to acquire high-purity CTCs from patient blood, while preserving their capacity to initiate tumors and metastasize, facilitating robust analysis of single CTCs. Using the system, the authors successfully purified CTCs from 70% of patients with primary and metastatic breast cancer and performed direct measurement of the gene expression in individual CTCs.

QDs are characterized by special optical properties, which enhance their usefulness in cancer cell detection [65]. Due to their high quantum yields, QDs are helpful in the detection of materials with low abundance. However, to enhance QD electrical characteristics, Pang et al. [52] hybridized ZnO NDs and g-C3N4 QDs to afford higher photoelectron transfer and separation efficiency. Due to the outstanding advantages, ZnO NDs and g-C3N4 QDs enjoyed the extended application, and a photocatalyzed renewable self-powered cytosensing device was presented on the basis of ZnO NDs@g-C3N4 QDs. Through conjugation to the membrane PTK7-specific aptamer Sgc8c, the device was used to detect CCRF-CEM cells (human acute lymphoblastic leukemia cells), which express PTK7. The results showed that the device offers better performance in terms of detection range, detection limit, selectivity, and reproducibility. It captured only CCRF-CEM cells (500 cell/mL) and no other cell types, such as HL-60, K562, and HeLa cells. The authors think that the device could be an effective platform for monitoring the progression of leukemia and shows great promise.

Polymer nanoparticles derived from various conductive hydrophobic polymers have been applied to produce nanoparticles with high quantum yields, photostability and that are nontoxic. Therefore, PD is ideal for CTC detection. Wu et al. [66] reported a strategy for semiconducting polymer dots (PDs) functionalization through entrapment of heterogeneous polymer chains into a single dot, which was facilitated by hydrophobic interactions, during nanoparticle formation. A few amphiphilic polymers with functional groups for subsequent covalent conjugation of biomolecules, such as streptavidin and immunoglobulin G (IgG), show co-condensation with most semiconducting polymers for modification and functionalization of a nanoparticle surface. The PDs bioconjugates were able to label cellular targets in an effective and specific manner, including a cell surface marker on human breast cancer cells, with no need to detect nonspecific binding. The authors demonstrated that the fluorescence exhibited by PD-labeled MCF-7 cells was 25 times higher than that of QD-labeled cells and 18 times higher than that of Alexa Fluor-labeled cells, according to flow cytometry analysis [67]. Based on the results, the authors believe that these ultrabright nanoparticles were successfully bioconjugated. Thus, this strategy provides a new opportunity for applying versatile semiconducting polymers to different fluorescence measurement methods in biomedicine and modern biology.

Upconversion nanoparticles (UCNPs) are usually selected for fluorescent labeling considering the ability to excite UCNPs with near-infrared (NIR) light to infrared (IR) light for generation of fluorescence emission in the visible region of the spectrum, leading to minimal background noise. Furthermore, applying NIR light as the excitation source prevents damage to normal tissues on one hand and allows deep tissue penetration on the other hand [68].

Shen et al. [69] described a simple method to conjugate multifunctional nanoparticles (MFNPs) assembled by the formation of various layers with a monoclonal anti-HER2 antibody and confirmed that the MFNPs exhibited the specific detection of breast cancer BT474 cells (biomarker HER2 positive) with a high signal-to-noise ratio. The MFNPs have an obvious core-shell structure of UCNP@Fe3O4@Au coated with anti-HER2 antibody and poly(ethylene glycol) (PEG) and exhibited an outstanding dispersity in different aqueous solutions and a high signal-to-noise ratio. The authors revealed that the photothermal effect exhibited a new high-localization feature at the single-cell level based under a continuous-wave near-IR laser. Using these nanoparticles, the authors exerted a photothermal effect at the single-cell level.

For differentiating types of cells and cancer states, the authors used AuNPs capped with ligands of different hydrophobicity and coated with green fluorescent protein (GFP). Because the capping ligands used showed different chemical structures, each AuNP-GFP complex was related to cancer cells to a different degree, considering cell membrane composition differences [70]. Magnetic biotargeting-multifunctional nanobioprobes (MBMNs) were used to detect and isolate a small subset of malignant cells from normal cells. CoFe2O4@BaTiO3 magnetoelectric nanoparticles distinguished different cancer cells from each other and from their normal counterparts through a magnetoelectric effect [71].

#### Detection through cell surface protein recognition

The main method to detect cancer cells relies on binding of nanoparticle probes conjugated with moieties (protein, short peptides, antibodies, oligonucleotide aptamers) to surface markers on cancer cells and on those entering cells and detecting genetic content. For the detection of cancer cells, such as CTCs, capture or isolation is the first and most important stage. Although the cell physical properties, such as size, deformability, and density, are sometimes used, capture is primarily based on the affinity of cell surface molecules on CTCs detected with materials such as antibodies or aptamers. Unique surface proteins on CTCs are the primary targets.

Since many studies have demonstrated that EpCAM is highly expressed on CTCs from many human malignancies, EpCAM can be used as a cell surface biomarker. Hence, anti-EpCAM molecules are often applied to screening of CTCs. CTCs undergoing EMT would cause inefficient positive sorting on the basis of EpCAM expression. Therefore, another approach is to find supplemental or replacement markers for EpCAM. Many cell surface markers, such as vimentin, androgen receptor, glycan, major vault protein (MVP), and fibroblast activation protein α (FAPα), have been studied for the detection of CTCs. However, a majority of these markers are only specific to certain cells, and many markers do not exist after CTCs experienced EMT. More mesenchymal CTCs can be seen in the metastatic stages of cancer, and thus, seeking proper EMT markers to evaluate prognosis and metastasis in cancer patients is important. Here, we compile the recently identified cell surface protein markers for detection of CTCs in different cancer types (Table 2).

**Table 2:** Cell surface protein markers for CTC detection

Marker	Type of cell	Type of cancer	Detection method	Reference
EpCAM	CTC	Colorectal Breast Head and neck	CellSearch	[72]
EpCAM and FRα	CTC	Non-small cell lung cancer	CellSearch	[73]
Glycan	CTC	Breast	Flow cytometry	[74]
Vimentin	CTC	Gastrointestinal	Confocal microscopy	[75]
	EMT CTC	Prostate	CellSearch	[76]
	CTC	Sarcoma	Flow cytometry	[77]
Vimentin+PD-L1	CTC	Colorectal, prostate cancer	Confocal microscopy	[78]
Synaptophysin	CTC	Castration resistant prostate cancer	CellSearch	[79]
Major vault protein	Mesenchymal and intermediate CTCs	Hepatocellular carcinomas	Flow cytometry	[80]
Androgen receptor	CTC	Metastatic breast cancer	Epic Sciences CTC platform	[81]
p75 Neurotrophin receptor+EpCAM	CTC	Esophageal squamous cell carcinoma	Flow cytometry	[82]
Carbonic anhydrase 9 and CD147	CTC	Clear cell renal cell carcinoma	NanoVelcro	[83]
Excision repair cross-complementation group 1	CTC	Platinum resistance ovarian cancer	Reverse-transcription PCR	[84]

#### Detection based on mRNA

In addition to the detection of extracellular nucleic acids, nanoparticles have also been developed as intracellular nucleic acid sensors. Seferos et al. [85] demonstrated that it is possible to use novel gold nanoparticle probes modified by oligonucleotides hybridized to complements labeled with a fluorophore as transfection agents and cellular “nanoflares” to detect mRNA in living cells. Nanoflares overcome many challenges in the creation of effective and sensitive intracellular probes and show a large signal-to-noise ratio and sensitivity to changes in the number of RNA transcripts in cells. Nanoflares, which show high orientation, dense oligonucleotide coating and can enter cells without the need for cytotoxic transfection agent s[86], are useful for detecting intracellular mRNA.

Meanwhile, researchers have developed nanoflares for simultaneous intracellular detection of various mRNA transcripts. In these multiplexed nanoflare studies, AuNPs functionalized with 2-3 DNA recognition strands and later hybridized with short complementary reporter strands were generated as nanoflares. For example, the use of multiplexed nanoflares to detect survivin in addition to actin has been investigated for normalizing nanoflare fluorescence differences in cellular uptake. Therefore, the technique is comparable with conventional qRT-PCR for quantification of intracellular mRNA but can be performed at the single live cell level.

In some cases, the nanoflare platform was expanded to quantify intracellular RNA and detect spatiotemporal localization in living cells [87]. In this work, β-actin-targeting nanoflares were incubated with HeLa cells and presented an obviously different intracellular distribution, exhibiting strong colocalization with mitochondria, which has not been previously demonstrated. Further, Smart-Flares were utilized for studying melanoma tumor cell heterogeneit y[88]. These Smart-Flares were able to quantify genomic expression at the single-cell level, thus expanding our knowledge of cancer and metastasis. Investigating the heterogeneity of cancer cells is crucial for identifying novel biomarkers for early cancer diagnosis.

Halo et al. [89] reported nanoflares, which were applied to capture live circulating breast cancer cells. These nanoflares could detect target mRNA in model metastatic breast cancer cell (MBC) lines in human blood and exhibited high recovery and fidelity reaching 99%. They also used nanoflares together with later cultured mammospheres to reimplant the retrieved live recurrent breast cancer cells into whole human blood. Only 100 live cancer cells could be detected per mL of blood. Relying on the NanoFlare technology, it was possible to simultaneously isolate and characterize intracellular live cancer cells from whole blood. The authors demonstrated the ability of nanoflares to collect CTCs for future culture and study. In addition, nanoflares contribute to the technology of combining intracellular markers with cell-surface markers for dually identifying putative CTCs. The combined method is likely to enhance the function of more platforms to specifically identify CTCs and subpopulations of CTCs. The authors think that nanoflares provide the first gene-based approach to detect, isolate, and characterize live cancer cells in the blood and are likely to contribute to cancer diagnosis, prognosis, and prediction, as well as personalized treatment.

Lee et al. reported an approach based on an elegant plasmonic nanoparticle network structure, generating a plasmon-coupled dimer able to detect single mRNA variants [90]. They applied the method to the detection and quantification of BRCA1 mRNA splice variants in vitro and in vivo. Two probes conjugated to nanoparticles were connected to the BRCA1 mRNA target in a sequence-specific manner, and as a result, the signal exhibited a spectral shift due to dimer formation. They demonstrated that their method is powerful and can successfully detect, quantify, and differentiate between different BRCA1 splice variants with single-copy sensitivity, thereby laying a foundation for quantitative, single-cell genetic profiling in the future.

### Nanotechnology for in vivo imaging

In addition to cancer diagnosis through ex vivo detection of cancer cells and biomarkers in liquid biopsy samples, identifying cancerous tissues in the body has many advantages in diagnosing and treating cancer. A proper nanoparticle probe for detecting cancer tissue should exhibit a long circulation time, be specific to tumor tissue and present low toxicity to nearby healthy tissue [7]. Current related studies have focused on nanoparticle probe accumulation in tumor tissue for diagnosing cancer in animal models, generally mouse models.

Nanoparticle probes can preferentially accumulate in tumor tissues through active or positive targeting, thereby allowing imaging and diagnosis of cancer in vivo [91]. Interactions between nanoparticles and blood proteins, uptake and clearance by the reticuloendothelial system (RES), penetration into solid tumors, and optimized active (vs passive) targeting for diagnosis of cancer constitute the main clinical application barriers. Fortunately, many developments related to these aspects have been achieved.

#### Passive targeting

By definition, passive targeting represents the preferential extravasation capacity of 10- to 150-nm nanoparticles from the bloodstream into tumor tissue. Because the tight junctions between endothelial cells in new blood vessels in tumors do not form properly, nanoparticles can preferentially accumulate in tumor tissue [92]. This form of passive nanoparticle entry into the tumor microenvironment is called the enhanced permeability and retention (EPR) effect, which was detected approximately 30 years ago by studying macromolecule transport into tumor tissues [93].

QDs are characterized by obvious photostability, tunable emission, and high quantum yield, contributing to their wide application in tumor tissue imaging through passive accumulation dependent on the EPR effect. Hong et al. [94] reported the use of a new NIR-II fluorophore, six-armed PEG-Ag_2_S QDs, for imaging of subcutaneous xenograft 4T1 murine tumors. They monitored how the NIR-II signal was distributed within the mice for a long period of time (up to 24-h post-injection (p.i.)) and observed that the NIR-II fluorescence of 6PEGAg_2_S QDs increased stably in the tumor region and decreased in the skin and other organs in the range of 30 min p.i. to 24 h p.i. The in vivo QD pharmacokinetics suggested extraordinary accumulation of 6PEG-Ag_2_S QDs in tumors (> 10% ID/gram, where % ID/gram denotes the concentration of the probe relative to the injected dose (ID) percentage per gram of tissue) through the EPR effect. They assert that imaging with these NIR-II QDs offered deep inner organ registration, dynamic tumor contrast, and rapid tumor detection.

Researchers have also applied AuNPs to in vivo tumor imaging through passive targeting. Lai et al. [95] reported that mercaptoundecanoic acid-coated AuNPs could identify and track primary glioma cells at the inoculation sites in mouse brains. Furthermore, these particles detected tumor-associated microvasculature in detail. In some cases, chitosan nanoparticles have been used for in vivo imaging through the EPR effect. Nam et al. [96] reported a tumor-targeting nanoparticle for use as an underlying multimodal imaging probe through optical/MR (MR: magnetic resonance) dual imaging based on self-assembled glycol chitosan. Through chemical modification and conjugation, they developed stable chitosan nanoparticles labeled with Cy5.5 and encapsulated by Gd(III) (Cy5.5-CNP-Gd(III)). The Cy5.5-CNP-Gd(III) were spherical, with a size of approximately 350 nm. According to cellular experiments, Cy5.5-CNP-Gd(III) were taken up in an effective manner, and distribution in the cytoplasm was observed. After administration via the tail vein of tumor-bearing mice, the nanoparticles localized in large numbers in the tumor, which was detected via noninvasive NIR fluorescence together with an MR imaging system. The authors propose that the unique characteristics of the glycol chitosan nanoparticles, such as blood stability, deformability, and quick cellular uptake, may greatly affect their in vivo tumor targeting ability, which relied on the EPR effect. Their results revealed that Cy5.5-CNP-Gd(III) could potentially be applied as an optical/MR dual imaging agent for detecting and treating cancer.

Nanoparticle size and shape affect the EPR effect. Therefore, these factors should be considered when designing nanoparticle probes for high tumor accumulation. Nanoparticles with a size of less than 10 nm can be quickly eliminated by the kidneys, minimizing their localization in tumor tissue [97]. Anisotropic particles exhibit an enhancement in circulation time, possibly because anisotropic nanoparticles are less likely to permeate endothelial gaps in the liver in the range of hundreds of nanometers to tens of micrometers. Silica-coated QDs of various thicknesses were used to explore the impact of nanoparticle size on tumor tissue accumulation [98]. The 12-nm QDs penetrated the tumor tissue with minimal hindrance, while the 60-nm QDs extravasated but remained in 10-μm blood vessels. By contrast, the 120-nm QDs showed no appreciable extravasation.

When nanoparticles contact a biological fluid, their surface will be become covered with a “corona” of biological macromolecules [99]. As serum proteins adsorb onto a nanoparticle surface (opsonization), the in vivo trafficking, uptake, and clearance of nanoparticles are greatly changed. Using PEG to coat a nanoparticle surface reduces nonspecific adsorption of serum proteins and minimizes protein corona formation, which increases the circulating time of the nanoparticle. PEGylation of various nanoparticles, such as AuNPs and QDs, results in a longer circulation time in the blood, as well as slow accumulation in the liver and spleen [100].

It is expected that nanotechnology-based imaging can improve the specificity and sensitivity of cancer diagnosis on the one hand and reduce toxicity on the other hand. Garrigue et al. [101] recently reported that harnessing nanoparticles and the “enhanced permeation and retention” (EPR) effect helped them develop an innovative nanosystem for positron emission tomography (PET) imaging. The system adopts a self-assembling amphiphilic dendrimer that retains various PET reporting units at terminals. This dendrimer was able to self-assemble into small uniform nanomicelles, which accumulated in tumors, allowing effective PET imaging. Due to the dendrimeric multivalence combined with the passive tumor targeting mediated by EPR, the nanosystem exhibited better imaging sensitivity and stronger specificity, with PET signal ratios that increased by approximately 14-fold in comparison with the clinical gold standard 2-fluorodeoxyglucose ([18F] FDG). Moreover, the dendrimer displayed an outstanding safety profile and good pharmacokinetics for PET imaging. The authors believe that their study contributes to the development of dendrimer nanosystems for effective and promising cancer imaging.

#### Active targeting

In addition to tumor imaging with the help of nanoparticle accumulation via passive targeting based on the EPR effect, scholars have implemented a large number of studies on recognition of receptors on the cell surface for active targeting of tumor tissues. Usually, these methods increase the number of nanoparticles delivered to tumor tissue in each unit time, thereby enhancing the sensitivity exhibited by in vivo tumor detection methods [102]. For the detection of tumors at an early stage with high contrast imaging, active tumor targeting achieves a better result than passive targeting that relies on the EPR effect.

Levenson and Nie reported antibody-conjugated QDs to target PSMA for active tumor targeting. The in vivo imaging results for 3 types of QD surface modifications were examined: (1) COOH groups, (2) PEG groups, and (3) PEG plus PSMA Ab (PEG-PSMA Ab). Consistent with the histological examinations, the COOH probe did not present any tumor signals, and only weak tumor signals were observed with the PEG probe (passive targeting), but the PEG-PSMA Ab-conjugated probe (active targeting) exhibited intense signals. The comparison confirmed the conclusion of more efficient and much quicker active targeting of tumors with a tumor-specific ligand compared with passive targeting in terms of tumor permeation, retention, and uptake [103].

A recent study showed the frequent application of peptides to active targeting of cancerous tissues in vivo. The RGD peptide is recognized by a receptor (integrin αvβ3) on the cell surface involved in cancer angiogenesis and metastasis and has been applied to the targeting of tumor tissue in vivo for diagnosis [104]. In one study, an iRGD-mediated and enzyme-induced precise targeting gold nanoparticle system (iRGD/AuNPs-A&C) was developed by simply co-administering a tumor-homing penetration peptide iRGD with a legumain-responsive aggregable gold nanoparticle. Intravenously injected compounds coupled to iRGD were bound to tumor vessels and then spread to extravascular tumor parenchyma, while traditional RGD peptides only transported cargo into blood vessels. iRGD homes to tumors through three steps: the RGD motif shows a mediating effect on the binding to αv integrins on the tumor endothelium, and then, a proteolytic cleavage imposes a binding motif for neuropilin-1, which regulates penetration into the cells. Conjugation to iRGD contributed to an obvious improvement in the sensitivity of the tumor imaging agents and the activity of the anti-tumor drug [34].

Previous studies reported Gd(3+)-DOTA and RGD (UCNP-Gd-RGD)-labeled upconversion nanoprobe for glioblastoma dual-modality imaging. To prepare UCNP-Gd-RGD, the amine-functional upconversion nanoparticle core is PEGylated, followed by Gd(3+) DOTA conjugation and RGD labeling. The colloidal stability of the obtained UCNP-Gd-RGD is improved and the cytotoxicity reduced compared with the UCNP core counterpart. Additionally, the UCNP-Gd-RGD presented an intense upconversion luminescence in the deep-red region and a 3-fold enhancement in T1 relaxivity compared with Gd(3+) DOTA. Considering the recognition between integrin αvβ3 receptors and UCNP-Gd-RGD, the nanoprobe exhibited specific binding to U87MG cells under confocal microscopy and quantification of ICP-MS. Furthermore, according to the UCNP-Gd-RGD in vivo upconversion fluorescence/MR imaging experiments together with the ex vivo analysis, subcutaneous U87MG tumor xenografts presented preferential retention [34].

One study reported a platform based on DNA that can be self-assembled into NIR-responsive NPs for cancer treatment. The platform has 3 different functional components: (1) complementary DNA strands, (2) gold nanorods (NRs) (50 nm × 10 nm), and (3) a polyethylene glycol (PEG) layer. The complementary DNA strands have sequential CG base pairs and offer some loading sites for doxorubicin (Dox), which is a model chemotherapeutic drug. The drug loading could be precisely tuned by changing the CG base pair number. One strand of DNA (called the capture strand), besides being a scaffold to carry the drug, can be used for capturing the gold NRs after being thiolated, and the complementary strand (called the targeting strand) can be used for specifically targeting cells after being preconjugated with ligand. Gold NRs are the model NIR light-to-heat transducers used for cancer thermotherapy and for denaturation of double-stranded DNA under NIR irradiation, as a result, loaded drugs are released at the target site for chemotherapy [105].

For CEA-overexpressing solid tumors, AMG 211 is a potentially interesting new bispecific T-cell engager (BiTE) antibody construct. AMG 211 was labeled with zirconium-89 (^89^Zr) or a fluorescent dye to evaluate its tumor-targeting properties. ^89^Zr-AMG211 microPET imaging can be complemented with ex vivo biodistribution and tracer integrity analysis. ^89^Zr-AMG211 showed dose-dependent CEA-specific tumor targeting and localization in viable tumor tissue. It can be used to clinically evaluate the in vivo AMG 211 behavior [106]. For example, ^89^Zr-AMG211 demonstrates specific tumor uptake in LS174T colorectal carcinoma xenografts, and microPET images revealed tumor uptake of ^89^Zr-AMG211 up to 24 h after injection, whereas the nontumor targeting BiTE antibody construct ^89^Zr-Mec14 did not show accumulation in LS174T xenografts.

Oseledchyk et al. reported a surface-enhanced resonance Raman scattering (SERRS) nanoparticle conjugated with folate receptor for in vivo imaging of xenograft SKOV-3 ovarian cancer cells transduced with green fluorescent protein and luciferase in a mouse model. This method was termed Topically Applied Surface-Enhanced Resonance Raman Ratiometric Spectroscopy (TAS3RS) and employed an effective ratiometric imaging approach with nontargeted SERRS-NP (nt-NP) and anti-FR-SERRS-NP (αFR-NP) multiplexing, successfully detecting tumor lesions in a murine model of human ovarian adenocarcinoma despite the size or localization of the tumor. TAS3RS can be used to detect microscopic residual tumors during surgery [107].

### Clinical trials of nanotechnology-based applications in cancer diagnosis

Nanotechnology for cancer diagnosis or detection has been extensively studied in the laboratory; however, clinical application is the ultimate destination of these studies. Currently, multiple nanotechnology-based cancer diagnosis approaches are in clinical trials (Table 3). For example, researchers at MSKCC and Cornell University have developed silica-hybrid nanoparticles (C-dots) for PET imaging of patients with metastatic melanoma or malignant brain tumors. These nanoparticles coupled with 124I-labeled cyclo-[Arg-Gly-Asp-Tyr] (cRGDY) peptides that are able to selectively bind to integrins can be used to probe tumor cells. In addition, another group of researchers (also from MSKCC) have developed fluorescent cRGDY C-dots (cRGDY-PEG-Cy5.5-C dots) for lymph node mapping, which can be used during surgery to visualize lymph nodes with cancer. As the research progresses, it is predictable that more and more nanotechnology-based cancer diagnostic methods will progress into the clinic.

**Table 3:** List of clinical trials of nanotechnology-based applications in cancer diagnosis

Title	Disease	Nanotechnology	Purpose	Phase	NCT number
Carbon Nanoparticles as lymph node tracer in rectal cancer after neoadjuvant radiochemotherapy	Rectal cancer	CNP (carbon nanoparticle)	Lymph node tracer	Not applicable	NCT03550001
Ferumoxytol-iron oxide nanoparticle magnetic resonance dynamic contrast-enhanced MRI	Head and neck cancer	Iron oxide nanoparticle	Imaging	Early Phase 1	NCT01895829
Application of carbon nanoparticles in laparoscopic colorectal surgery	Colorectal tumor	CNP	Tumor localization and lymph node mapping	Not applicable	NCT03350945
Sentinel lymph node mapping in endometrial cancer	Endometrial neoplasms	CNP	Lymph node mapping	Not applicable	NCT03778255
Sentinel lymph node mapping in cervical cancer	Uterine cervical neoplasms	CNP	Lymph node mapping	Not applicable	NCT03778268
Targeted silica nanoparticles for real-time image-guided intraoperative mapping of nodal metastases	Head and neck cancer Melanoma Breast cancer Colorectal cancer	Fluorescent cRGDY-PEG-Cy5.5-C dots	Imaging/Localization	Phase 1/Phase 2	NCT02106598
Nanochip technology in monitoring treatment response and detecting relapse in participants with diffuse large B-cell lymphoma	Lymphoma	Immuno-tethered lipoplex nanoparticle	Monitoring/detection	Not applicable	NCT03656835
Diagnosis of gastric lesions with Na-nose	Stomach diseases/Gastric cancer	Nanosensors	Diagnosis		NCT01420588
PET imaging of patients with melanoma and malignant brain tumors using a 124I-labeled cRGDY silica nanomolecular particle tracer: a microdosing study	Metastatic melanoma/malignant brain tumors	124I-cRGDY-PEG-dots	Localization	Not applicable	NCT01266096
Imaging of patients with malignant brain tumors using 89Zr-cRGDY ultrasmall silica particle tracers: a phase 1 microdosing study	Brain cancer	89Zr-DFO-cRGDY-PEG-Cy5-C' dots	PET imaging	Phase 1	NCT03465618

### Challenges in clinical translation

Although there has been much promising progress in nanotechnology-based cancer diagnosis, only a few examples have advanced to clinical trials [108]. To accelerate the translation of nanotechnology into clinical applications, many challenges need to be addressed.

The first challenge in nanotechnology-based cancer diagnosis is reliability. To be applied in the clinic, it is essential to obtain reliable and quantitative detection results. Many factors can affect NP-based detection signals, including nonspecific binding of NP probes, aggregation and unfit detection conditions [109]. Fluctuations in the signals can also be attributed to complicated body fluid compositions. From a clinical validation perspective, assay reliability and reproducibility need to be extensively investigated in large clinical sample pools before NP-based assays can reach the clinical application.

The second challenge lies in large-scale production of nanoprobes that are highly sensitive, highly reproducible, and have long-term storage stability at an acceptable cost [110]. The production of most of the current nanoprobes is performed under highly optimized conditions in labs; however, it is still a big challenge to produce these probes in batches. Because the shape, size, composition, charge, and surface coating of nanoprobes vary, the detection results also greatly vary. To minimize batch-to-batch changes, the synthesis steps and nanoprobe functionalization must be simplified. In addition, nanoprobes may tend to aggregate during storage. Moreover, the cost-effectiveness of developing a nanotechnology-based platform must be taken into consideration.

The third challenge is to develop NP-based devices with high sensitivity and that are easy to handle and cost-efficient. Most NP-based assays were prepared in academic laboratories, and many assays are unrealistic for clinical translation. For example, complicated confocal Raman microscopes were used to implement most studies based on SERS but are rarely present in hospitals or clinical laboratories. Successful development of NP-based POC (point of care) devices will greatly facilitate clinical application of nanotechnology in cancer diagnosis.

The fourth challenge is the possible toxicity of nanoparticles induced by their systemic administration. This challenge is mostly related to NP-based imaging in vivo. To apply novel nanoparticle probes to in vivo imaging, the possible toxicity of these nanoparticles should be assessed. The properties of nanoparticles (such as shape, size, charge, surface chemistry, targeting ligands, and composition) can influence their toxicity. In addition, the biodistribution, biodegradability, and pharmacokinetic properties of nanoparticles should be considered.

## Conclusion

**Fig. 2:**
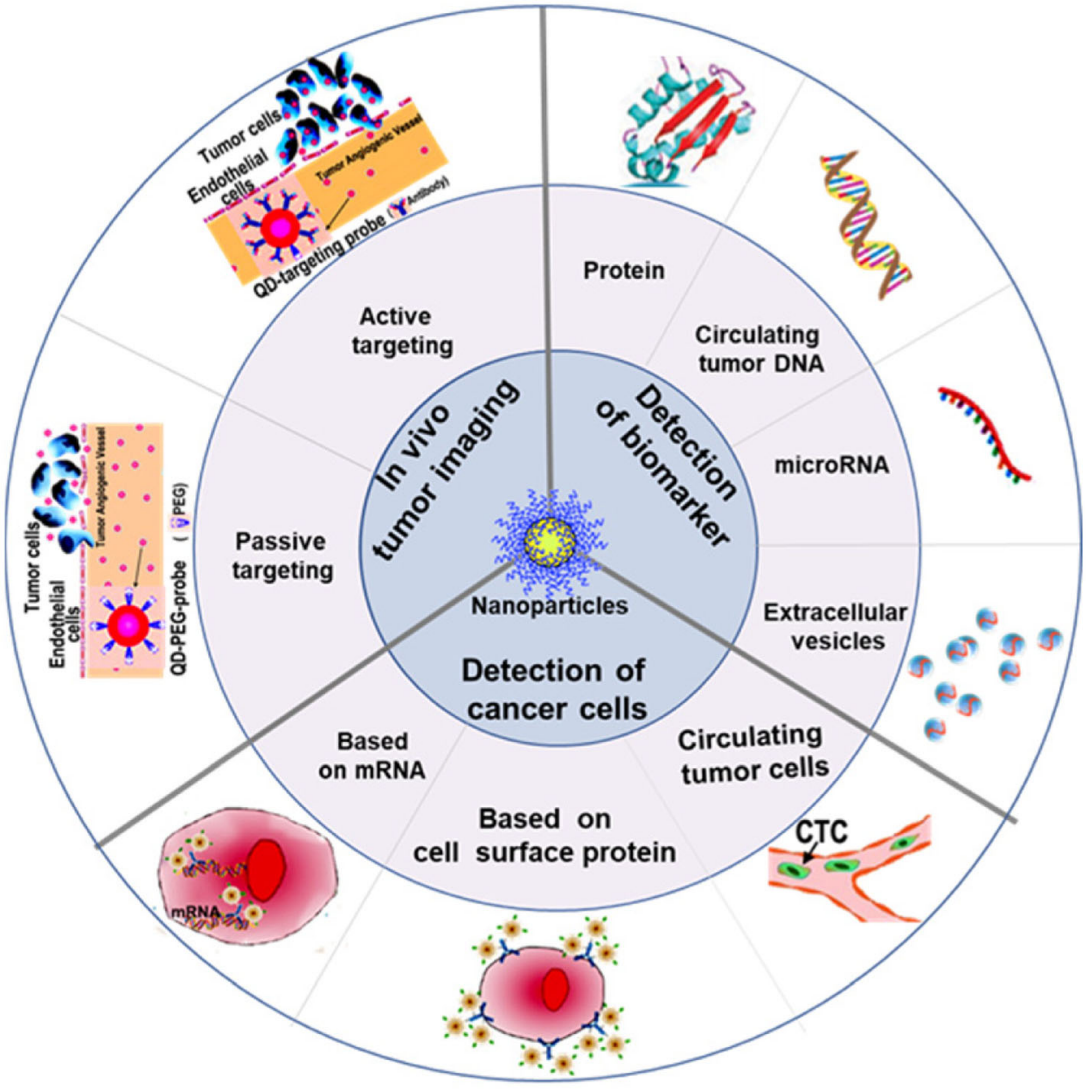
Schematic illustration of nanotechnology applications in cancer diagnosis

The recent progress in nanotechnology-based application in cancer diagnosis has been summarized in this review (Fig. 2). In the past 10 years, many efforts have been made to develop assays for cancer diagnosis based on nanotechnology. Compared with the currently available cancer diagnostics in the clinic, a variety of NP-based assays showed improvement in terms of selectivity and sensitivity or offered entirely new capacities that could not be achieved with traditional approaches. These advances will improve the survival rate of cancer patients by enabling early detection. In addition, these advances could be used to monitor cancer progress in response to treatment, which may contribute to the development of better strategies for cancer treatment.

Over the last decade, great progress has been made in the field of nanotechnology-based cancer diagnosis, and our understanding in this field has greatly improved. Although only a few NP-based assays have advanced to clinical trials, with close collaboration among researchers, engineers, and clinicians, nanotechnology-based cancer diagnosis is poised to move into the clinic in the near future. With its high sensitivity, specificity, and multiplexed measurement capacity, nanotechnology provides great opportunities to improve cancer diagnosis, which will ultimately lead to an improved cancer patient survival rate.

## Abbreviations

AuNPs: Gold nanoparticles: BiTE: Bispecific T-cell engager BLI: Bioluminescence imaging: CA 19-9: Carbohydrate antigen 19-9 CEA: Carcinoembryonic antigen CT: Computed tomography CTCs: Circulating tumor cells ctDNA: Circulating tumor DNA Dox: Doxorubicin DSN: Duplex-specific nuclease EPR: Enhanced permeation and retention Evs: Extracellular vesicles MRI: Magnetic resonance imaging NC: Nanocluster NPs: Nanoparticles NSE: Neuron-specific enolase PDAC: Pancreatic ductal adenocarcinoma PDs: polymer dots PEG: Polyethylene glycol PET: Positron emission tomography PL: photoluminescence PSMA: Prostate-specific membrane antigen QDs: Quantum dots SERRS: Surface-enhanced resonance Raman scattering sPLA-2: Secreted phospholipase A2 ssDNA: Single-stranded DNA SWV: Square wave stripping voltammetry TAS3RS: Topically Applied Surface-Enhanced Resonance Raman Ratiometric Spectroscopy UCNPs: Upconverting nanophosphors, Upconversion nanoparticles ZnO: Zinc oxide
